# Impact of 1-methylcyclopropene and controlled atmosphere storage on polyamine and 4-aminobutyrate levels in “Empire” apple fruit

**DOI:** 10.3389/fpls.2014.00144

**Published:** 2014-04-10

**Authors:** Kristen L. Deyman, Carolyne J. Brikis, Gale G. Bozzo, Barry J. Shelp

**Affiliations:** Department of Plant Agriculture, University of GuelphGuelph, ON, Canada

**Keywords:** abiotic stress, apple fruit, controlled atmosphere storage, 4-aminobutyrate (GABA), 1-methylcyclopropene (1-MCP), high performance liquid chromatography, polyamines

## Abstract

1-Methylcyclopropene (1-MCP) delays ethylene-meditated ripening of apple (*Malus domestica* Borkh.) fruit during controlled atmosphere (CA) storage. Here, we tested the hypothesis that 1-MCP and CA storage enhances the levels of polyamines (PAs) and 4-aminobutyrate (GABA) in apple fruit. A 46-week experiment was conducted with “Empire” apple using a split-plot design with four treatment replicates and 3°C, 2.5 kPa O_2_, and 0.03 or 2.5 kPa CO_2_ with or without 1 μL L^-1^ 1-MCP. Total PA levels were not elevated by the 1-MCP treatment. Examination of the individual PAs revealed that: (i) total putrescine levels tended to be lower with 1-MCP regardless of the CO_2_ level, and while this was mostly at the expense of free putrescine, large transient increases in soluble conjugated putrescine were also evident; (ii) total spermidine levels tended to be lower with 1-MCP, particularly at 2.5 kPa CO_2_, and this was mostly at the expense of soluble conjugated spermidine; (iii) total spermine levels at 2.5 kPa CO_2_ tended to be lower with 1-MCP, and this was mostly at the expense of both soluble and insoluble conjugated spermine; and (iv) total spermidine and spermine levels at 0.03 kPa were relatively unaffected, compared to 2.5 kPa CO_2_, but transient increases in free spermidine and spermine were evident. These findings might be due to changes in the conversion of putrescine into higher PAs and the interconversion of free and conjugated forms in apple fruit, rather than altered *S*-adenosylmethionine availability. Regardless of 1-MCP and CO_2_ treatments, the availability of glutamate showed a transient peak initially, probably due to protein degradation, and this was followed by a steady decline over the remainder of the storage period which coincided with linear accumulation of GABA. This pattern has been attributed to the stimulation of glutamate decarboxylase activity and inhibition of GABA catabolism, rather than a contribution of PAs to GABA production.

## INTRODUCTION

**FIGURE 1:**
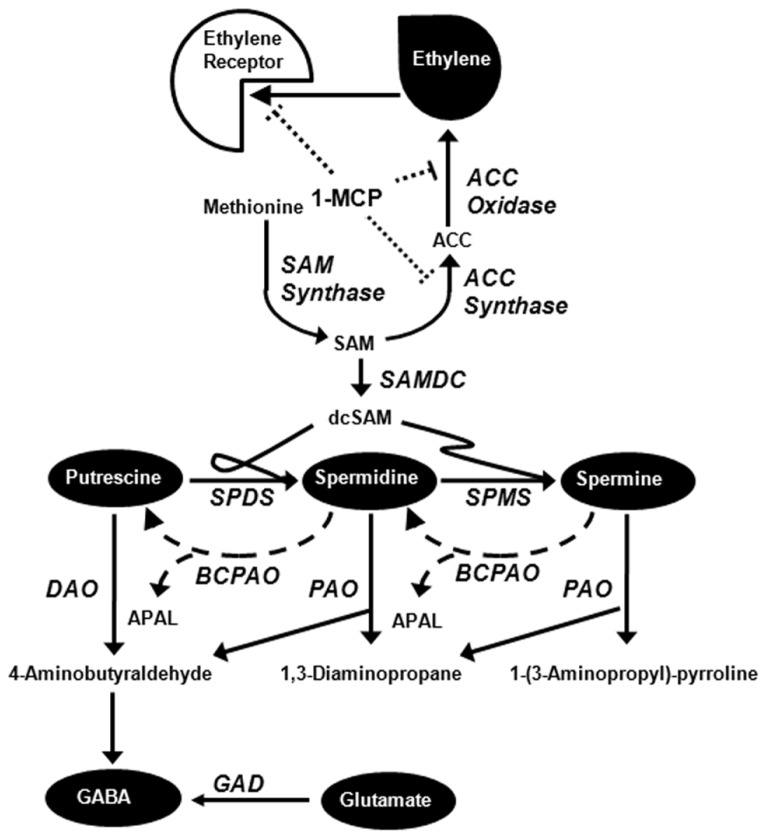
**Simplified diagram of metabolic relationships among ethylene, polyamines, and GABA [adapted from Shelp et al. (2012c)].** 1-MCP inhibits ethylene binding to its receptor and the expression of both ACC synthase and ACC oxidase (see text for detailed explanation). Abbreviations: ACC, 1-aminocyclopropane-1-carboxylic acid; APAL, 3-aminopropionaldehyde; BCPAO, back-conversion polyamine oxidase; dcSAM, decarboxylated *S*-adenosylmethionine; DAO, diamine oxidase; GABA, 4-aminobutyrate; GAD, glutamate decarboxylase; 1-MCP, 1-methylcyclopropene; PAO, polyamine oxidase; SAM, *S*-adenosylmethionine; SAMDC, SAM decarboxylase; SPDS, spermidine synthase; SPMS, spermine synthase.

Over the last decade, 1-methylcyclopropene (1-MCP) has been adopted by the apple industry as a means of delaying ethylene-mediated fruit ripening and senescence, especially in combination with storage under controlled atmosphere (CA) conditions (i.e., 0–3°C, 2–2.5 kPa O_2_, 2–4 kPa CO_2_; DeEll et al., 2008; Fawbush et al., 2008; Watkins, 2008). 1-MCP inhibits ethylene binding and production in apple fruit held at ambient or chilling temperature, and reduces the expression of genes responsible for ethylene biosynthesis (Dal Cin et al., 2006; Pang et al., 2006; Vilaplana et al., 2007; **Figure 1**). Under commercial CA conditions, these findings are accompanied by a decline in the level of 1-aminocyclopropane-1-carboxylic acid (ACC), and the level of its precursor *S*-adenosylmethionine (SAM) is not directly linked to the rate of ethylene production and does not appear to be limiting (Bulens et al., 2012).

*S*-adenosylmethionine is essential for conversion of putrescine (Put) into the higher polyamines (PAs) spermidine (Spd) and spermine (Spm), as well the production of ethylene (**Figure 1**). During the ripening of a bulky fruit such as tomato, there is an inverse relationship between the production of higher PAs and ethylene (Lasanajak et al., 2014), 1-MCP treatment inhibits autocatalytic ethylene production without affecting SAM levels (Van de Poel et al., 2013), and decreased levels of ethylene in RNAi-1-aminocyclpropane-1-carboxylate synthase fruits enhance PA levels and upregulate PA biosynthesis genes (Gupta et al., 2013). Research with non-bulky fruits such as rice grain also suggests interaction between PAs and ethylene in regulating plant growth and in response to environmental stress (Chen et al., 2013). Notably, there is a transient increase in the level of Put only, and no changes in the level of Spd, Spm, or total PAs in apple fruit stored at 24°C, even though the expression of only one of two SAM decarboxylases (SAMDCs) is repressed (Pang et al., 2006).

During CA storage 4-aminobutyrate (GABA) accumulates in apple fruit (Deewatthanawong and Watkins, 2010; Lee et al., 2012; Trobacher et al., 2013a), but it is uncertain whether this GABA is derived from PAs or glutamate (Shelp et al., 2012c; **Figure 1**). Notably, the levels of both PAs and GABA can be stimulated by abiotic stresses such as chilling, O_2_ deficiency and elevated CO_2_, conditions that are the basis of CA storage (Alcázar et al., 2010; Bitrián et al., 2012; Shelp et al., 2012a,b,c; Trobacher et al., 2013a).

Here, we tested the hypothesis that 1-MCP and CA storage enhances the levels of PAs and GABA in apple fruit imposed by abiotic stresses associated with CA storage. Since 1-MCP has been shown to increase the incidence of CO_2_-induced physiological injury in “Empire” fruit during CA storage (Watkins, 2006; Fawbush et al., 2008; Jung and Watkins, 2011), we utilized two levels of CO_2_ (2.5 and 0.03 kPa).

## MATERIALS AND METHODS

### APPLE SOURCE AND CONTROLLED ATMOSPHERE STORAGE TRIALS

Sixteen boxes (~20 kg each) of apple (*Malus domestica* Borkh. cv. Empire) fruit were harvested in the morning from a commercial orchard within 15 km of Simcoe, Ontario on 29 September 2011, and immediately transported to our post-harvest facility at the University of Guelph. On arrival, four fruit were randomly chosen, frozen as quickly as is practical in liquid N_2_ and stored at -80°C for future metabolite analysis. Then the boxes were divided into two treatments without or with 1 μL L^-1^ 1-MCP (SmartFresh^SM^, AgroFresh Inc., Spring House, PA, supplied in sealed polybags) and stored at approximately 23°C for 1 day.

A split-plot design was used to eliminate the possibility of chamber effects. Fruit were stored in two random chambers that were set at 3°C; within each chamber, two rooms were supplied with 2.5 kPa O_2_ and either 2.5 kPa or 0.03 kPa CO_2_, giving a total of four rooms, each containing fruit treated without or with 1-MCP. The four rooms in each chamber were treated as biological replicates in this nested design.

About 90 fruit were stored in individual boxes in the CA rooms, which were sealed and flushed with 99 kPa N_2_. When the O_2_ concentration declined to 2.5 kPa, N_2_ flushing was replaced with CO_2_ until 2.5 or 0.03 kPa CO_2_ was achieved. Four fruit were collected from each box at eight different time points over the storage period (17 days, and 4, 8, 10, 14, 23, 33, and 46 weeks) following harvest, frozen in liquid N_2_ and stored at -80°C prior to metabolite analysis. Fruit were also collected for assessment of fruit quality, but these data will be reported elsewhere.

### METABOLITE COMPOSITION OF WHOLE APPLE FRUIT

#### Polyamine analysis

For each treatment replicate, four whole frozen apples were cryogenically pulverized, taking care to ensure that the apple tissue did not thaw during the procedure (Trobacher et al., 2013a). PAs were essentially extracted and analyzed as described by Smith and Davies (1985) and Shiozaki et al. (2000). Briefly, 100 mg of fine frozen powder (comprised of 25 mg from each of the four apple subsamples in each treatment replicate) was homogenized in cold 5% perchloric acid (PCA, 100 mg mL^-1^), placed on ice for 30 min and then spiked with 10 μL of the internal standard 1,6 hexanediamine (0.25 nmol μL^-1^; Fisher Scientific, Whitby, ON, Canada). The sample was centrifuged for 20 min at 14,000 *g* at room temperature, and the supernatant was transferred to a 2-mL microfuge tube and maintained on ice for 30 min. The pellet was extracted again in 1 mL of 5% PCA and treated as above. For analysis of free and conjugated soluble polyamines, the two supernatants were combined and dried in vacuo at 60°C or under a stream of filtered air at room temperature, then redissolved in 0.4 mL of 5% PCA. One half of this extract was transferred to a 5-mL amber reacti-vial and derivatized for analysis of free PAs as described below. The remaining 0.2 mL was hydrolyzed with HCl as described below before derivatization. The soluble conjugated soluble polyamines were estimated as the difference between the hydrolyzed and original supernatants.

The final pellet was resuspended in 0.2 mL of 1 M NaOH and centrifuged for 5 min at 10,000 *g*. The supernatant was transferred to a 2-mL amber Eppendorf tube and hydrolyzed with 0.2 mL of 12 M HCl at room temperature for 18 h. The sample was then dried as above, resuspended in 0.2 mL of 5% PCA and derivatized for analysis of conjugated insoluble polyamines.

A 0.6 mL aliquot of dansyl chloride (Sigma Aldrich, Oakville, ON, Canada) in acetone (7.5 mg mL^-1^ acetone) was added to a 0.2-mL aliquot of the various PCA extracts. Then 0.3 mL of a saturated sodium carbonate solution was added with brief vortexing to give a pH of 10. The mixture was placed in a 60°C waterbath for 30 min (Gennaro et al., 1988). A 0.1-mL aliquot of proline (0.1 *g* mL^-1^; Sigma Aldrich) was added to the solution to remove excess dansyl chloride, and then the mixture was incubated in the dark for 15 min at room temperature. Dansylated PAs were extracted from the mixture by adding 0.5 mL toluene (Fisher Scientific, Whitby, ON, Canada) and vortexing for 1 min. Then, 0.4 mL was taken from the organic layer and dried under filtered air for 30 min. The dry residue was dissolved in 0.2 mL methanol and stored at 4°C in the dark for up to 2 weeks.

Dansylated PAs were passed through 0.45 um syringe filter and 20-μL aliquots injected onto a reverse-phase column (Agilent Zorbax ODS 5 μm, 4.6 mm × 150 mm) linked to an Agilent 1100 HPLC system (Allan and Shelp, 2006). Initially, 60% methanol was provided for 4 min at a flow rate of 1.5 mL min^-1^, followed by a linear increase to 95% methanol over 10 min and then 95% methanol for a further 5 min. The PAs were quantified with a fluorescence detector set at excitation and emission wavelengths of 254 and 500 nm, respectively (Gennaro et al., 1988). Although other peaks might appear on the chromatogram, they eluted before all dansylated PAs of interest, allowing for good resolution of Put (RT = 12.6 min), 1,6 hexanediamine (RT = 13.8 min), Spd (RT = 16.4 min), and Spm (RT = 18.5 min). Each treatment replicate was considered to be the average of the four subsamples.

The linearity of the standard curves up to 120 pmol for Put, Spd, Spm and 1,6 hexanediamine (Sigma Aldrich, Oakville, ON, Canada) was not affected by the addition of frozen apple powder tissue to the extraction procedure. The recovery of the internal standard across apple samples was approximately 60%; all samples were corrected for the actual loss during preparation. Furthermore, reverse-phase HPLC analysis of the PA standards and apple samples showed baseline separation of all PAs of interest. For routine analysis, a suite of external standards (80 pmol each) was run every sixth sample. The detection limit for the overall method was approximately 0.10 nmol *g*^-1^ fresh mass (FM).

#### Amino acid analysis

The amino acid composition of each apple was determined essentially as described previously (Allan and Shelp, 2006). Briefly, 1 *g* of the fine frozen powder was ground in four volumes of 30 *g* L^-1^ sulfosalicylic acid using a chilled mortar and pestle and fine silica sand, and 1.0 mL of the solution was centrifuged. The supernatant was adjusted to neutrality with NaOH, and then passed through a 0.45-μm syringe filter prior to on-line derivatization with *o*-phthalaldehyde. Aliquots (0.5 μL) of the supernatant were analyzed by reverse-phase HPLC. Each treatment replicate was considered to be the average of the four subsamples analyzed and each mean was the average of four treatment replicates. A suite of external amino acid standards (125 pmol each) derived from protein hydrolysate and the individual amino acids GABA, asparagine and glutamine were run every sixth sample.

#### Statistical analysis of data

All statistical analyses were conducted using SAS 9.2 at the α = 0.05 level (SAS Institute Inc., Cary, NC, USA). Replicate room effects were analyzed using analysis of variances (ANOVAs; proc mixed method), which partitioned variance into the fixed effects (1-MCP, temperature, CO_2_, storage time) and their interactions, and the random effect of chambers. In cases where interactions were significant (*P* ≤ 0.05), treatment means were compared within weeks (slice option) over the period from 17 days to 46 weeks using a Fisher’s protected least significant difference test. For experiments where interactions were significant (*P* ≤ 0.05), data were pooled across repeated measures to determine differences among treatments and over storage period using the Tukey’s test. All data were arcsine square root transformed to ensure a normal distribution of variance and the treatment means were back-transformed for presentation.

## RESULTS

**FIGURE 2:**
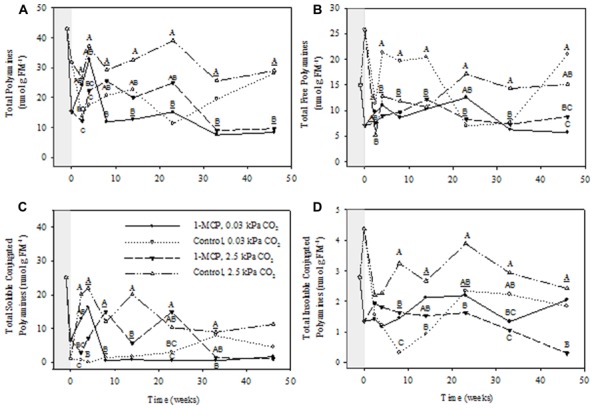
**Effects of 1-MCP and elevated CO_**2**_ on total **(A)**, total free **(B)**, total soluble conjugated **(C)**, and total insoluble conjugated **(D)** polyamines in “Empire” fruit during controlled atmosphere storage.** With the exception of the beginning and end of 1-MCP treatment (-1 day to 0 week, the shaded area), all data are mean estimates of four storage replicates. Different letter groupings indicate significant differences between treatments within weeks (*P* ≤ 0.05). Underlined letters indicate a shared letter for overlapping data; where letters are absent at a time point, there were no significant differences.

**FIGURE 3:**
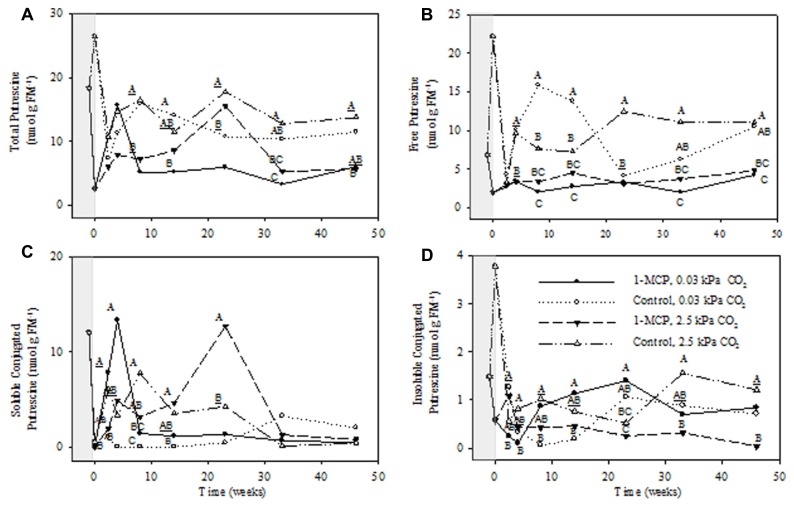
**Effects of 1-MCP and elevated CO_**2**_ on total **(A)**, free **(B)**, soluble conjugated **(C)**, and insoluble conjugated **(D)** putrescine in “Empire” fruit during controlled atmosphere storage.** With the exception of the beginning and end of 1-MCP treatment (-1 day to 0 week, the shaded area), all data are mean estimates of four storage replicates. Different letter groupings indicate significant differences between treatments within weeks (*P* ≤ 0.05). Underlined letters indicate a shared letter for overlapping data; where letters are absent at a time point, there were no significant differences.

**FIGURE 4:**
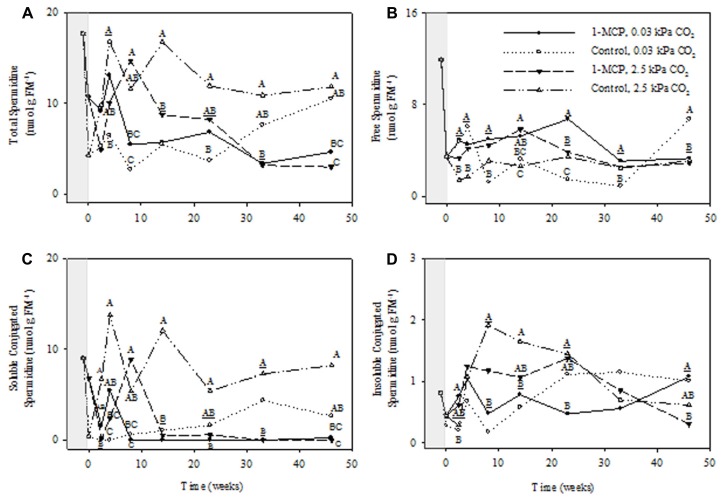
**Effects of 1-MCP and elevated CO_**2**_ on total **(A)**, free **(B)**, soluble conjugated **(C)**, and insoluble conjugated **(D)** spermidine in “Empire” fruit during controlled atmosphere storage.** With the exception of the beginning and end of 1-MCP treatment (-1 day to 0 week, the shaded area), all data are mean estimates of four storage replicates. Different letter groupings indicate significant differences between treatments within weeks (*P* ≤ 0.05). Underlined letters indicate a shared letter for overlapping data; where letters are absent at a time point, there were no significant differences.

**FIGURE 5:**
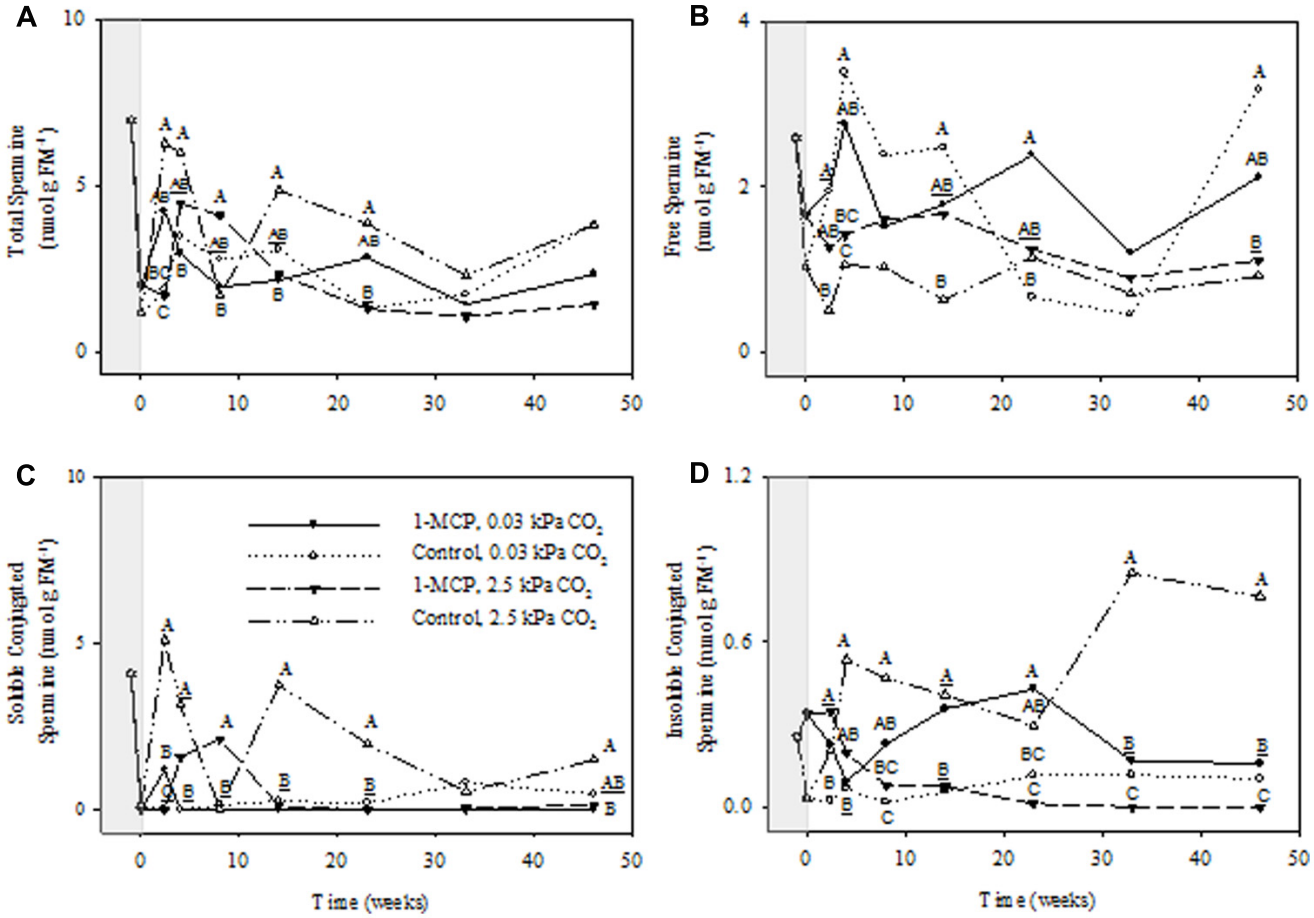
**Effects of 1-MCP and elevated CO_**2**_ on total **(A)**, free **(B)**, soluble conjugated **(C)**, and insoluble conjugated **(D)** spermine in “Empire” fruit during controlled atmosphere storage.** With the exception of the beginning and end of 1-MCP treatment (-1 day to 0 week, the shaded area), all data are mean estimates of four storage replicates. Different letter groupings indicate significant differences between treatments within weeks (*P* ≤ 0.05). Underlined letters indicate a shared letter for overlapping data; where letters are absent at a time point, there were no significant differences.

Total apple PAs consisted of free, soluble conjugated, and insoluble conjugated forms, with free and soluble conjugated forms being at much higher concentrations than the insoluble conjugated forms (**Figures 2A–D**). The concentrations of all forms fluctuated considerably during the 1-MCP treatment and the initial collection periods at 17 days and 4 weeks, but were much steadier from 8 to 46 weeks. Therefore, statistical analysis of the main effects and interactions was conducted for the entire treatment period, whereas detailed comparisons between the 1-MCP treatments were made over the 8–46 week period. Furthermore, the total PAs were comprised primarily of total Put and total Spd, followed by total Spm (**Figures 3–5**, panel A). The concentrations of individual PAs consisted mainly of the free form, followed closely by the soluble conjugated form, and more distantly by the insoluble conjugated form (**Figures 3**–**5**, panels B–D). Notably, the concentrations of the free forms tended to fluctuate less than the conjugated forms.

The concentrations of total PAs showed significant storage time, CO_2_ and 1-MCP main effects, as well as storage time × CO_2_, 1-MCP × CO_2_, and storage time × CO_2_ × 1-MCP interactions (*P* ≤ 0.05, ANOVA table not shown). During the period from 8 to 46 weeks, the total PA concentrations in 1-MCP-treated fruit at 2.5 kPa CO_2_ were, with the exception of 8 and 23 weeks, 33–61% of those in control fruit (i.e., not treated with 1-MCP), whereas at 0.03 kPa CO_2_ they were 29–39% of those at 33 and 46 weeks (**Figure 2A**). The concentrations of total free PAs showed significant storage time and CO_2_ main effects, as well as storage time × 1-MCP, and storage time × 1-MCP × CO_2_ interactions (*P* ≤ 0.05, ANOVA table not shown). At 2.5 kPa CO_2_ the total free PA concentrations in 1-MCP-treated fruit were 49–51% of those in control fruit at 23 and 33 weeks, whereas at 0.03 kPa CO_2_ they were, with the exception of weeks 23 and 33, 27–52% of those in control fruit (**Figure 2B**). The concentrations of total soluble conjugated PAs showed significant CO_2_ and 1-MCP main effects, as well as CO_2_ × 1-MCP, storage time × CO_2_ × 1-MCP interactions (*P* ≤ 0.05, ANOVA table not shown). At 2.5 kPa CO_2_, the concentrations of total soluble conjugated PAs in 1-MCP-treated fruit were similar to those in control fruit, whereas at 0.03 kPa CO_2_ they were 6% of that in the control at 33 weeks (**Figure 2C**). The concentrations of total insoluble conjugated PAs showed significant CO_2_ and 1-MCP main effects, and storage time × 1-MCP, CO_2_ × 1-MCP interactions (*P* ≤ 0.05, ANOVA table not shown). At 2.5 kPa CO_2_ the concentrations of total insoluble conjugated PA concentrations in 1-MCP- treated fruit were, with the exception of week 14, 13–57% of those in control fruit, whereas at 0.03 kPa CO_2_ they were 130–350% greater at 8 and 14 weeks (**Figure 2D**).

The concentrations of total Put displayed significant CO_2_ main effects, but no interactions (*P* ≤ 0.05, ANOVA table not shown). The total Put concentrations at 2.5 and 0.03 kPa CO_2_ in 1-MCP-treated fruit were 41–44% and 32–37% of those in control fruit at 8, 33, and 46 weeks and at 8, 14, and 33 weeks, respectively (**Figure 3A**). There were significant storage time and CO_2_ main effects on the concentrations of free Put, as well as storage time × CO_2_ × 1-MCP interaction (*P* ≤ 0.05, ANOVA table not shown). At 2.5 kPa CO_2_ the free Put concentrations in 1-MCP-treated fruit were 25–44% of those in control fruit at 23, 33, and 46 weeks, whereas at 0.03 kPa CO_2_ they were, with the exception of week 23, 13–40% of the controls (**Figure 3B**). The concentrations of soluble conjugated Put showed significant 1-MCP and CO_2_ main effects, as well as storage time × 1-MCP and storage time × CO_2_ × 1-MCP interactions (*P* ≤ 0.05, ANOVA table not shown). At 2.5 kPa CO_2_ the concentrations of soluble conjugated Put in 1-MCP-treated fruit were 200% greater than those in control fruit at 23 weeks, whereas at 0.03 kPa CO_2_ there were no significant differences among treatments (**Figure 3C**). The concentrations of insoluble conjugated Put showed significant CO_2_ main effects, as well as storage time × CO_2_, storage time × 1-MCP, CO_2_ × 1-MCP and storage time × CO_2_ × 1-MCP interactions (*P* ≤ 0.05, ANOVA table not shown). At 2.5 kPa CO_2_ the concentrations of insoluble conjugated Put in 1-MCP-treated fruit were 3–20% of those in control fruit at 33 and 46 weeks, whereas at 0.03 kPa CO_2_ they were 490–1420% greater than controls at 8 and 14 weeks (**Figure 3D**).

The total Spd concentrations displayed significant storage time, CO_2_ and 1-MCP main effects, as well as storage time × CO_2_, storage time × 1-MCP, and CO_2_ × 1-MCP interactions (*P* ≤ 0.05, ANOVA table not shown). At 2.5 kPa CO_2_ total Spd concentrations in 1-MCP-treated fruit were 25–52% of those in control fruit at 14, 33, and 46 weeks, whereas at 0.03 kPa CO_2_ there were no significant differences between treatments (**Figure 4A**). Free Spd concentrations showed significant storage time, CO_2_, and 1-MCP main effects, as well as storage time × CO_2_, storage time × 1-MCP, and storage time × CO_2_ × 1-MCP interactions (*P* ≤ 0.05, ANOVA table not shown). At 2.5 kPa CO_2_ free Spd concentrations in 1-MCP-treated fruit were 121% greater than those in control fruit at 14 weeks, whereas at 0.03 kPa CO_2_ they were 230–350% greater at 8, 23, and 33 weeks and 49% of the control at 46 weeks (**Figure 4B**). The concentrations of soluble conjugated Spd displayed CO_2_ and 1-MCP main effects, and storage time × CO_2_, CO_2_ × 1-MCP and storage time × CO_2_ × 1-MCP interactions (*P* ≤ 0.05, ANOVA table not shown). At 2.5 kPa CO_2_ the concentrations of soluble conjugated Spd in 1-MCP-treated fruit never exceeded 4% of those at 14, 33, and 46 weeks of storage, whereas at 0.03 kPa CO_2_, they were undetectable at 33 weeks of storage (**Figure 4C**). The concentrations of insoluble conjugated Spd showed significant storage time and 1-MCP main effects and storage time × 1-MCP interactions (*P* ≤ 0.05, ANOVA table not shown). At 2.5 and 0.03 kPa CO_2_, there were no significant differences between the 1-MCP treatments (**Figure 4D**).

The concentrations of total Spm showed significant storage time and CO_2_ main effects, as well as CO_2_ × 1-MCP and storage time × CO_2_ × 1-MCP interactions (*P* ≤ 0.05, ANOVA table not shown). At 2.5 kPa CO_2_ total Spm concentrations in 1-MCP-treated fruit were 140% greater than those that in control fruit at 8 weeks, but 34% of the control at 23 weeks (**Figure 5A**). There were no differences between treatments at 0.03 kPa CO_2_. The concentration of free Spm showed significant storage time and 1-MCP main effects (*P* ≤ 0.05, ANOVA table not shown). At 2.5 kPa CO_2_ there was no significant differences between the treatments, whereas at 0.03 kPa CO_2_ the free Spm concentration was 260% greater in 1-MCP-treated fruit than that in control fruit at 23 weeks (**Figure 5B**). There were significant CO_2_ and 1-MCP main effects, as well as storage time × CO_2_, CO_2_ × 1-MCP and storage time × CO_2_ × 1-MCP interactions, for concentrations of soluble conjugated Spm (*P* ≤ 0.05, ANOVA table not shown). At 2.5 kPa CO_2_ the concentration of soluble conjugated Spm in 1-MCP-treated fruit was much greater than that in control fruit at 8 weeks, but less than 3% of those at 14 and 23 weeks, whereas at 0.03 kPa CO_2_ there were no differences between 1-MCP treatments (**Figure 5C**). The concentrations of insoluble conjugated Spm showed significant CO_2_ and 1-MCP main effects, as well as storage time × CO_2_, CO_2_ × 1-MCP interactions (*P* ≤ 0.05, ANOVA table not shown). At 2.5 kPa CO_2_ the concentrations of insoluble conjugated Spm in 1-MCP-treated fruit never exceeded 18% of those in control fruit over the 8- to 46-week period; however, at 0.03 kPa CO_2_ the concentrations were 280–1250% greater at 8, 14, and 23 weeks (**Figure 5D**).

**FIGURE 6:**
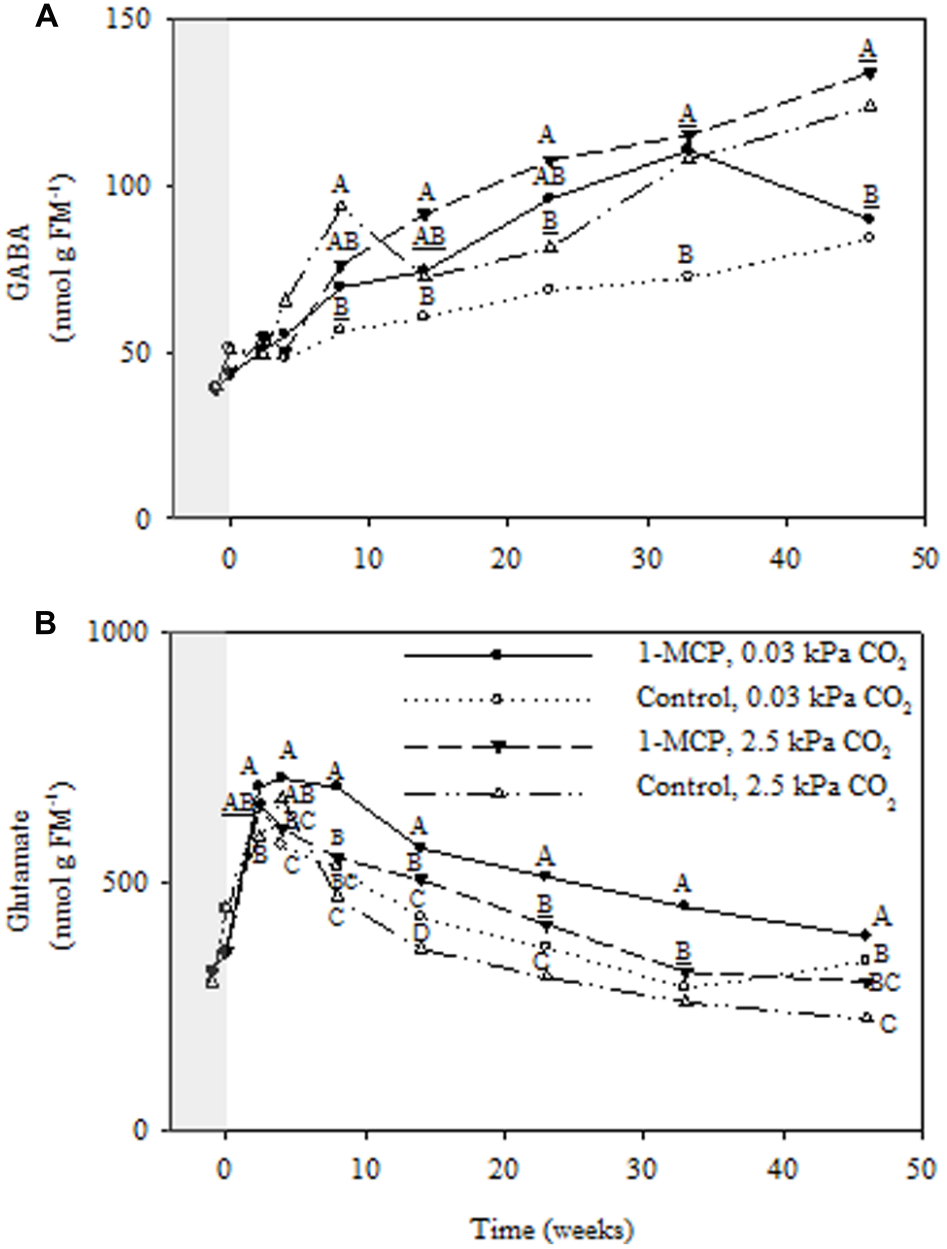
**Effects of 1-MCP and elevated CO_**2**_ on GABA **(A)** and glutamate **(B)** levels in “Empire” fruit during controlled atmosphere storage.** With the exception of the beginning and end of 1-MCP treatment (-1 day to 0 week, the shaded area), all data are mean estimates of four storage replicates. Different letter groupings indicate significant differences between treatments within weeks (*P* ≤ 0.05). Underlined letters indicate a shared letter for overlapping data; where letters are absent at a time point, there were no significant differences.

Regardless of 1-MCP and CO_2_ treatments GABA accumulated in a linear fashion over the storage period (**Figure 6A**), whereas glutamate declined after an initial rise (**Figure 6B**). Notably, fruit receiving 2.5 kPa CO_2_ and 1-MCP accumulated twice as much GABA (~2 nmol *g*^-1^ FM week^-1^) as fruit receiving 0.03 kPa CO_2_ only, accounting for approximately one half of the decline in glutamate over the same period. The GABA concentrations displayed significant storage time, CO_2_ and 1-MCP main effects, as well as storage time × 1-MCP interactions (*P* ≤ 0.05, ANOVA table not shown). At 2.5 and 0.03 kPa CO_2_ the GABA concentrations in 1-MCP-treated fruit were 19 and 25% greater, respectively, than those in control fruit at 33 weeks (**Figure 6A**). Glutamate showed significant storage time, CO_2_ and 1-MCP main effects, as well as storage time × 1-MCP, CO_2_ × 1-MCP interactions (*P* ≤ 0.05, ANOVA table not shown). At 2.5 kPa CO_2_ glutamate concentrations in 1-MCP-treated fruit were 34–38% greater than those in control fruit at 14 and 23 weeks, whereas at 0.03 kPa CO_2_ they were 8–57% greater than those in control fruit over the storage period (**Figure 6B**).

## DISCUSSION

Both PAs and GABA appear to function in various physiological processes such as stress responses and growth and development (Alcázar et al., 2010; Fincato et al., 2012; Moschou et al., 2012; Shelp et al., 2012b,c). The presence and interconversion of free forms of Put, Spd and Spm have been the focus of PA research, but soluble (i.e., bound to small molecules such as phenolic acids) and insoluble (i.e., bound to macromolecules such as nucleic acids and proteins) conjugated forms also exist. The ratios between free and conjugated PAs vary among plant species (Bagni and Tassoni, 2001), and some evidence exists for interconversion between free and conjugated PAs (Bassard et al., 2010), which could influence our interpretation of the impact of 1-MCP and CA on PAs and possibly GABA in apple fruit (**Figure 1**). Interestingly, it has been suggested that the stress hormone abscisic acid contributes to the conversion of conjugated forms of PA to the free forms (Ben Hassine et al., 2009).

In the present study, the concentrations of free and conjugated forms of PAs were determined in ripening 1-MCP-treated “Empire” apple fruit over a 46-week storage period under CA conditions. Initially, there were marked fluctuations in the concentrations of these PAs as the fruit acclimated to the shift in environmental conditions. Steady-state levels were evident after approximately 8 weeks of storage. It is well known that 1-MCP treatment should inhibit the autocatalytic production of ethylene in apple fruit (Pang et al., 2006; DeEll et al., 2008; Fawbush et al., 2008; Jung and Watkins, 2011), reduce the expression of genes involved in ethylene biosynthesis (Dal Cin et al., 2006; Pang et al., 2006; Vilaplana et al., 2007), and decrease the level of ACC (Bulens et al., 2012). However, we found no evidence for elevated levels of total PAs, Put, Spd, or Spm during the steady-state period (8–46 weeks) in response to 1-MCP treatment; indeed the levels tended to be lower regardless of CO_2_ (**Figures 2**–**5**), suggesting that the availability of SAM for PA biosynthesis in apple fruit was not influenced by the 1-MCP treatment. These findings are consistent with previous studies of ripening apple and tomato fruits, which suggest that the requirement for SAM in PA biosynthesis is not limited by the requirement in ethylene biosynthesis (Van de Poel et al., 2013; Lasanajak et al., 2014), and suggest an unknown biochemical or transcriptional mechanism, possibly altering the PA biosynthetic rates from glutamate (Alcázar et al., 2010; Majumdar et al., 2013), was responsible for the lower total PA levels in 1-MCP-treated fruit under CA storage. However, these findings are at odds with the generally accepted view that PAs, especially Spd and Spm, accumulate in respond to abiotic stress (Groppa and Benavides, 2008; Bassard et al., 2010; Shelp et al., 2012c), which could be due to a variety of reasons: that view is generally based on free PA levels, rather than total PAs; apple fruit experience multiple stresses during CA storage; and, ripening apple fruit, like tomato fruit (Mattoo et al., 2010), are at a terminal developmental stage.

1-Methylcyclopropene application changed the levels of individual PAs and the relative proportions of free and conjugated forms. Total Put levels tended to be lower with 1-MCP regardless of the CO_2_ level, and while this was mostly at the expense of free Put, large transient increases in soluble conjugated Put were also evident (**Figure 3**). Total Spd levels tended to be lower with 1-MCP, particularly at 2.5 kPa CO_2_, and this was mostly at the expense of soluble conjugated Spd (**Figure 4**). Total Spm levels at 2.5 kPa CO_2_ tended to be lower with 1-MCP, and this was mostly at the expense of both soluble and insoluble conjugated Spm (**Figure 5**). Overall, total Spd and Spm levels at 0.03 kPa were relatively unaffected, compared to 2.5 kPa CO_2_, but transient increases in free Spd and Spm were evident. Thus, 1-MCP treatment reduced the accumulation of individual PAs with elevated CO_2_ under CA conditions; however, there was a general decrease and increase in the ratio of free:conjugated forms for Put and Spd plus Spm, respectively. The accumulation of PAs was unaffected under low CO_2_, but there was some evidence for accumulation of conjugated Spd and Spm. Since total PA levels declined with 1-MCP (**Figure 2**), this differential response of Put and Spd plus Spm in the partitioning between free and conjugated forms could not be attributed to altered SAM availability. The response might be attributed, at least in part, to changes in the conversion and back-conversion of PAs, and the interconversion of free and conjugated forms in apple fruit, although the activities of polyamine oxidases (PAOs) could be limited by the low O_2_ status of intact apple fruit stored under CA conditions (Ho et al., 2011; Shelp et al., 2012c).

Glutamate serves a precursor for biosynthesis of both GABA and PAs, raising the possibility that GABA in CA-stored apple fruit can be derived directly from glutamate or indirectly from free Put or free Spd (**Figure 1**). Regardless of 1-MCP and CO_2_ treatments, the availability of glutamate showed a transient peak initially, probably due to protein degradation (Magné et al., 1997; Sugimoto et al., 2011), and this was followed by a steady decline over the remainder of the storage period which coincided with accumulating GABA (**Figure 6**). This pattern could be attributed due to the stimulation of glutamate decarboxylase (GAD) activities via bound Ca^2+^/calmodulin or lower cytosolic pH (Shelp et al., 2012a; Trobacher et al., 2013b), and product inhibition of GABA transaminase activity (Clark et al., 2009) due to the restricted activity of succinic semialdehyde dehydrogenase activity under O_2_ deficiency (Busch and Fromm, 1999; Shelp et al., 1999). GABA accumulation was lowest at 0.03 kPa CO_2_ and noticeably greater with either 1-MCP or 2.5 kPa CO_2_. These findings are consistent with previous research showing that GABA accumulates in tissues of “Empire” apple fruit during CA storage with elevated CO_2_ and is then catabolized when the fruit are transferred to ambient conditions (Deewatthanawong and Watkins, 2010; Lee et al., 2012; Trobacher et al., 2013a). Relatively higher levels of free Put and Spd than free Spm are also consistent with studies of other species and tissues (Mattoo et al., 2010). Interestingly, the changing levels of Spd seemed to be more highly correlated than those of Put with the rate of GABA production regardless of the 1-MCP and CO_2_ treatments, suggesting that Spd could be in steady-state equilibrium with GABA. It has been argued that changes in O_2_ availability and cellular redox balance in apple fruit stored under CA conditions would directly influence the activity of 4-aminobutyraldehyde dehydrogenase, as well as diamine oxidases (DAO), thereby restricting GABA formation from both Spd and Put (Shelp et al., 2012c). Unfortunately, pool sizes do not indicate the flux through a pathway, mutants of the metabolic routes for GABA and PAs are not readily available for apple, and radiolabelled precursors cannot be supplied to intact apples without perturbing the internal gaseous environment, which would most certainly affect GABA formation from glutamate. Useful information about the relative contributions of glutamate and PAs to GABA production in intact apple fruit could be gained via a combination of metabolite and gene transcript analyses. Recently, use of the DAO inhibitor aminoguanidine suggested that approximately 30% of the GABA accumulated in fava bean seed germinating under hypoxia is derived from PAs (Yang et al., 2013). Notably, the free levels of the three PAs, especially Put, increased, unlike the case reported here, and this was accompanied by some loss of GAD activity, which was attributed to growth inhibition.

While uncertainty continues regarding the relationship between PA and ethylene biosynthesis in fruits, especially those exposed to multiple abiotic stresses, our research indicates that the requirement for SAM in PA biosynthesis in CA-stored apple fruit was probably not limited by the requirement in ethylene biosynthesis. Moreover, the differential response of PA partitioning between free and conjugated forms could be attributed to changes in the interconversion of free and conjugated forms and the conversion and back-conversion of PAs. Also, the data could be interpreted as preliminary evidence for a relationship between Spd level and GABA production, but it is argued that the biochemical reactions involved would be limited by the *in vivo* O_2_ level, and that elevated GAD activity and product inhibition of GABA transaminase activity would be responsible for GABA accumulation in these fruit.

## Conflict of Interest Statement

This research was funded in part by AgroFresh Inc., the manufacturer of SmartFresh^SM^.
